# The kinetics of antibody binding to *Plasmodium falciparum *VAR2CSA PfEMP1 antigen and modelling of PfEMP1 antigen packing on the membrane knobs

**DOI:** 10.1186/1475-2875-9-100

**Published:** 2010-04-19

**Authors:** Lars M Joergensen, Ali Salanti, Tina Dobrilovic, Lea Barfod, Tue Hassenkam, Thor G Theander, Lars Hviid, David E Arnot

**Affiliations:** 1 Centre for Medical Parasitology, Department of International Health, Immunology & Microbiology, Faculty of Health Sciences, University of Copenhagen and Department of Infectious Diseases, Copenhagen University Hospital (Rigshospitalet), CSS Øster Farimagsgade 5, Building 22 & 23, Postbox 2099, 1014 Copenhagen K, Denmark; 2 Nano-Science Centre, Department of Chemistry, Faculty of Science, University of Copenhagen, Copenhagen K, Denmark; 3 Institute of Immunology & Infection Research, School of Biology, University of Edinburgh, West Mains Road, Edinburgh, EH9 3JT, UK

## Abstract

**Background:**

Infected humans make protective antibody responses to the PfEMP1 adhesion antigens exported by *Plasmodium falciparum *parasites to the erythrocyte membrane, but little is known about the kinetics of this antibody-receptor binding reaction or how the topology of PfEMP1 on the parasitized erythrocyte membrane influences antibody association with, and dissociation from, its antigenic target.

**Methods:**

A Quartz Crystal Microbalance biosensor was used to measure the association and dissociation kinetics of VAR2CSA PfEMP1 binding to human monoclonal antibodies. Immuno-fluorescence microscopy was used to visualize antibody-mediated adhesion between the surfaces of live infected erythrocytes and atomic force microscopy was used to obtain higher resolution images of the membrane knobs on the infected erythrocyte to estimate knob surface areas and model VAR2CSA packing density on the knob.

**Results:**

Kinetic analysis indicates that antibody dissociation from the VAR2CSA PfEMP1 antigen is extremely slow when there is a high avidity interaction. High avidity binding to PfEMP1 antigens on the surface of *P. falciparum*-infected erythrocytes in turn requires bivalent cross-linking of epitopes positioned within the distance that can be bridged by antibody. Calculations of the surface area of the knobs and the possible densities of PfEMP1 packing on the knobs indicate that high-avidity cross-linking antibody reactions are constrained by the architecture of the knobs and the large size of PfEMP1 molecules.

**Conclusions:**

High avidity is required to achieve the strongest binding to VAR2CSA PfEMP1, but the structures that display PfEMP1 also tend to inhibit cross-linking between PfEMP1 antigens, by holding many binding epitopes at distances beyond the 15-18 nm sweep radius of an antibody. The large size of PfEMP1 will also constrain intra-knob cross-linking interactions. This analysis indicates that effective vaccines targeting the parasite's vulnerable adhesion receptors should primarily induce strongly adhering, high avidity antibodies whose association rate constant is less important than their dissociation rate constant.

## Background

Antibody responses to parasite-encoded, variable erythrocyte surface antigens (VSA) are a major component in the natural acquisition of immunity to *Plasmodium falciparum *malaria [1-3]. Biosensors, capable of real-time measurement of the strength of molecular interactions, can be used to measure the kinetics of the antibody binding to the parasite antigen [4] and study the specific mechanisms of how antibodies act against infection [5].

Multi-domain PfEMP1 adhesion receptors are targets for host antibody during malaria infection [6-8]. Both IgG [6,9] and IgM [10] specifically bind purified PfEMP1 antigens. Non-specific IgG [11] and IgM [12] binding to *Plasmodium falciparum*-infected erythrocytes (IE) has also been reported, IgM binding being *via *the Cμ4 domain [13]. Antibody responses to *P. falciparum *erythrocyte surface antigens are initiated at a low parasitaemia and class switching from IgM to IgG occurs as the response is boosted by parasite replication [14,15]. Convalescent phase serum antibodies from recovering malaria patients can agglutinate parasites isolated during the previous clinical attack [16]. Cross-reactive antibodies binding malaria parasites from other infections are seen, but broadly reactive sera are rare [17,18].

Electron microscopy (EM) indicates that antibodies bind to the IE surface at the knob protrusions [19-21]. The response is directed against VSAs [1,22,23], but capping of knobs by antibody has not been observed in either EM or fluorescence microscopy (FM) using live IE [20,24]. Neither the binding kinetics nor the avidity of these interactions, *i.e*. the total binding strength of the multiple antibody-antigen interactions, have been measured for this or any other malaria antibody-antigen interaction.

Therefore, a Quartz Crystal Microbalance (QCM) biosensor was used to analyse monoclonal antibody binding to the VAR2CSA PfEMP1 antigen and carry out a kinetic analysis of binding between human anti-PfEMP1 antibodies and recombinant fragments of the VAR2CSA PfEMP1 antigen, under flow conditions. Having immobilized antigen, and antibody in the flow solution, is a more realistic model of the *in vivo *adhesion reaction than the reciprocal arrangement often used to estimate the 'pure' affinity of antibody-antigen reactions. This configuration also permits estimation of the avidity component [25].

To construct molecular models of the context in which the antibody-PfEMP1 reaction occurs on knob structures, these were first visualized at low resolution, using confocal optical microscopy, then at higher resolution, using atomic force microscopy (AFM) to obtain realistic parameters for structural modeling and consideration of the molecular distances involved in the interaction between epitopes on antigens and paratopes on antibodies.

## Methods

### Parasite culture and selections

*Plasmodium falciparum *clones 3D7 and FCR3 were cultured in group O erythrocytes using a modified Trager-Jensen procedure [20]. Cultures were genotyped using PCR primers targeting MSP2 and GLURP and tested for mycoplasma using the MycoAlert^® ^Detection Kit (Lonza). FCR3 cells expressing the *var2csa *gene and VAR2CSA protein were pre-selected on BeWo cells [26]. 3D7 IE expressing PfEMP1 were selected using streptavidin-coated Dynabeads^® ^and biotin-labelled antibodies, to capture parasites reacting with surface-binding antibodies [27].

### Antisera and chip-immobilized recombinant antigen

Rabbit antisera and human monoclonal antibodies targeting the VAR2CSA PfEMP1 protein have been described [28,29]. Affinity-purified rabbit antisera against baculovirus-expressed PfEMP1 VAR4 (from 3D7 ch.4 DBL4, PfD1235w) and VAR5 (from 3D7 ch.11 CIDR, Pf11_0008), each encoded by a different Group A-type *var *genes [9,30], were used in IFA. Recombinant proteins were purified and characterized as previously described [28-30]. Soluble protein was attached to quartz crystal sensor surfaces as described below.

### Confocal microscopy

A TE 2000-E Nikon Eclipse confocal microscope with 100× (N.A. 1.4) Apoplan oil immersion lens was used [24]. Images were captured using the EZ-C1 Gold imaging system (version 3.30), saved in IDS test format and processed using Adobe Photoshop^® ^software for sharpening edges, contrast and light adjustment. Images show the 5 μm size bar calculated by the EZ-C1 software.

### Quartz crystal microbalance

Attana Series 100 biosensors measured molecular interactions in flow, in real time and without labeling. Treated quartz crystal sensor surfaces were positioned in the flow cell with one side of the crystal exposed to continuous flow of protein. As sample reaches the sensor surface, binding of molecules to the crystal surface is detected as a decrease in the resonance frequency of the quartz crystal. The mass bound to the surface was calculated using the Sauerbrey equation [31], with a frequency shift of 1 Hz corresponding to a bound mass of around 4.4 ng/cm^2^

### Quartz crystal preparation

Gold plated, 10 MHz, AT-cut quartz crystals were purchased from Attana AB (Stockholm). The gold plates were pre-treated either to generate carboxyl groups or with a polystyrene coating. The surface was housed in a cylindrical flow chamber with a volume of 1.25 μl (height 50 μm, diameter 5.64 mm). All buffers and solutions were sterile filtered (0.2 μm) before use.

Carboxylated surfaces were used for coupling to amino groups of the protein to be immobilized. The surface was pre-wetted with high quality water (18.2 MΩcm) for 1 h, before insertion into the biosensor housing. The chip surface was allowed to stabilise in a flow of 10 mM HEPES (2- [4-(2-hydroxyethyl) piperazin-1-yl] ethanesulfonic acid) pH 7.0 running buffer. Following stabilisation, the surface was activated by freshly prepared 0.2 M EDC (1-ethyl-3-(3-dimethylaminopropyl) carbodiimide), 0.05 M Sulfo-NHS (N-hydroxy-sulfosuccinimide), for 5 minutes. Protein solutions to be immobilized onto the chip surface were diluted in 10 mM acetic acid pH 5.0. Protein was used at concentrations between 5-50 μg/ml, depending on the surface density required, and added by injection into the flow for 5 minutes, after which any remaining active sites were blocked by a 1 M ethanolamine solution pH 8.0 for 5 minutes.

Polystyrene surfaces were used to adsorb proteins. Protein was adsorbed *ex situ *for three hours at a concentration of 250 μg/ml in a 10 mM acetic acid 50 mM NaCl solution at pH 5.0. The kinetic analysis was performed with a running buffer containing 1 g/l BSA.

### IgG binding and kinetic analysis

Binding was carried out either at room temperature or 37°C (when using the thermo-controlled later version of the Attana 100), using PBS pH 7.4 as the running buffer. After the baseline was stabilised, mock binding runs using running buffer were performed to demonstrate that the stable base line signal was unaffected by the injection process. The buffer baseline signal was subtracted from all measurements. After testing at the chosen concentrations, re-test injections were carried out using the first concentrations tested, to estimate damage to the sensor surface in the course of the experiment. Kinetic data was processed and curves fitted to either a simple 1:1 binding model using software package Scrubber 2 (BioLogic Software Pty Ltd, Campbell, Australia), or a heterogeneity model using ClampXP (Tom Morton and David Myszka, version 3.50). The softwares use the Levenberg-Marquardt method for nonlinear sum of squares error minimization.

### Atomic force microscopy

Magnetically purified *P. falciparum *trophozoite and schizont infected red blood cells were washed and then spread on a glass slide and air dried prior to AFM scanning. The AFM (tapping mode) images of the dried but unfixed IE were recorded in air under ambient conditions with a scan speed of 1 Hz. AFM images were recorded with an Asylum MFP-3D, using a silicon tip (Olympus) with a spring constant of 2 N/m and a resonant frequency of 70 Hz.

## Results

### Visualizing antibody binding to the erythrocyte surface and the antibody-mediated agglutination reaction

**Figure 1:**
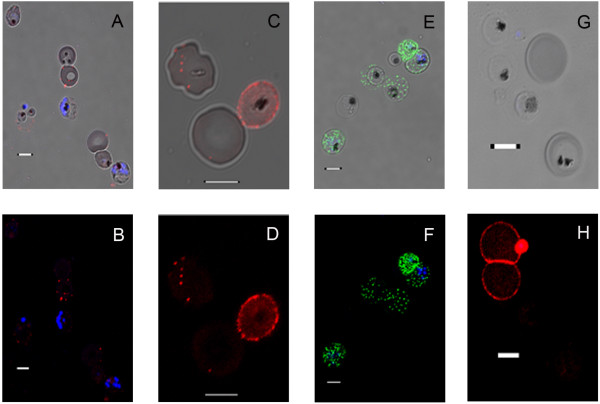
**Patient plasma IgG and anti-PfEMP1 IgG binding to IE**. IE incubated with goat anti-human IgG conjugated to Alexa^®^568 (red) or goat anti-rabbit IgG Alexa^®^488 (green). **A**. Antibody selected IE (3D7) incubated with 1:50 diluted Tanzanian child's acute-phase malaria serum. The DIC images of IE show DAPI stained parasite nuclei (blue) and spots of membrane-bound IgG (Alexa^®^568). **B**. Fluorescence image of Figure 1A. **C**. Higher magnification DIC image of live IE (3D7) and uninfected erythrocytes, incubated with 1:100 diluted convalescent plasma from the same child. **D**. Fluorescence image of Figure 1C. **E**. DIC images of 3D7, incubated with rabbit antiserum against a recombinant Var4 PfEMP1, detected with goat anti-rabbit IgG (Alexa^®^488). **F**. Fluorescence image of Figure 1E. **G**. DIC and DAPI (blue) fluorescence image of immune serum-agglutinated and unagglutinated erythrocytes. IE (FCR3) selected by panning on Dynabeads coated with IgG from a pool of semi-immune Tanzanian children's sera (note DAPI-staining merozoite invading the upper IE). **H**. Agglutination of two IE by serum used in Figures 1C and 1D, detected with goat anti-human IgG Alexa^®^568. Two infected and one uninfected erythrocytes are unagglutinated and largely unstained. The invading merozoite is stained by serum IgG. Live trophozoites often do not take up DAPI but such IE can be identified by pigment or serum staining (Figure 1E). Scale bars 5 μm.

Figure 1 shows the binding phenotypes of fluorescently labeled human IgG antibodies to IE. Figures 1A and 1B show IE incubated with plasma from the acute phase of a child's malaria attack. Figures 1C and 1D show IE binding plasma antibodies from a sample taken during the convalescence of the child. The 3D7 parasites were pre-selected for binding to anti-PfEMP1 antibodies [32]. Figures 1E and 1F show FCR3 parasites stained with an anti-VAR4 PfEMP1 antisera [30]. IE show relatively small amounts of punctate staining with the acute infection serum. Punctate staining with convalescent serum is more intense, indicating maturation of an antibody response targeting concentrations of antigen at particular spots on the IE membrane (compare Figures 1B and 1D).

Figures 1E and 1F show that, using a live staining and purified, specific anti-PfEMP1 antiserum, similar, but more delicately punctate patterns of IE surface staining [20] are obtained compared to crude patient sera. Figures 1G and 1H illustrate the capacity [33] of immune serum to mediate agglutination of IE. Antigenic specificity of this adhesion reaction [16] is seen, as those FCR3 parasites expressing particular PfEMP1 antigens [34] which do not bind serum IgG, remain unagglutinated. IE expressing recognized PfEMP1 antigens can thus be agglutinated by cross-linking IgG antibodies. However, the binding strengths of these interactions are not known.

### Measuring the strength of binding between anti-PfEMP1 antibodies and a PfEMP1 antigen

**Figure 2:**
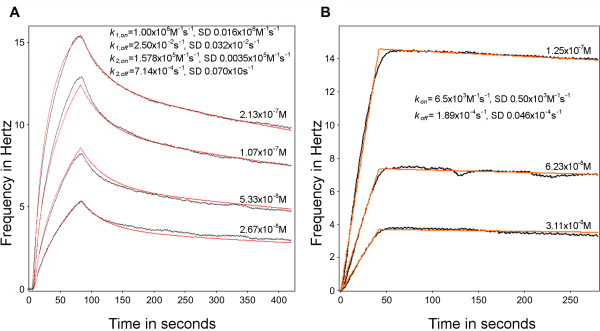
**Association and dissociation of a weakly binding antibody to VAR2CSA DBL5ε**. The frequency change (Hz) as a function of time (s), shows both the association and dissociation phase of the PAM 4.7 antibody binding to the DBL5ε domain of the VAR2CSA PfEMP1. Black lines indicate data; red lines indicate fitted curves (see Methods) and the calculated rate constants are given on the sensorgrams. **A**. PAM 4.7, in flow, reacting with VAR2CSA DBL5ε immobilized on a polystyrene surface, tested at the four concentrations shown. **B**. PAM 4.7, in flow, reacting with VAR2CSA DBL5ε, immobilized on a carboxyl surface and tested at three concentrations with a lower density of surface-immobilized antigen.

Biosensors can measure anti-PfEMP1 antibody PfEMP1 antigen kinetics and binding strengths and thus give a better understanding of the molecular interactions observed in immunofluorescence. The sensorgrams shown in Figure 2 show the binding of the PAM 4.7 monoclonal antibody [29] to a DBL5ε domain fragment of VAR2CSA measured using a QCM biosensor. A 40.1 kDa recombinant-produced fragment (339a.a. long), containing the DBL5ε domain of the VAR2CSA PfEMP1 antigen [35] was bound to either a polystyrene or a carboxyl surface of the quartz crystal. Attachment was detected by a negative resonant frequency shift, which has been converted into more easily analysed positive values in all Figures.

Figure 2A shows binding to antigen adsorbed to a polystyrene surface and Figure 2B shows binding to antigen immobilized *via *a carboxyl group. Resonant frequency changes over time indicate association and dissociation of the antibody to and from its epitope on the VAR2CSA DBL5ε antigen. Figure 2 shows that as the concentration of antibody increases, so does the initial rate of binding, essentially a trivial observation, not to be confused with the rate constants (*k*_*on *_and *k*_*off*_), which are concentration-independent properties of binding. In Figure 2A, using adsorption to polystyrene as the method of immobilizing antigen, relatively complex biphasic binding kinetics were seen, probably reflecting the situation where some antibody molecules bind two immobilized epitopes, while others only bind one. Thus two of each class of rate constants are given for the reaction. In Figure 2B, with higher concentrations of antigen covalently bound to the surface, simpler kinetics were observed. As in the experiments shown in Figure 2A, the actual reaction is not a simple 1:1 binding, since there are two Fabs per IgG molecule. However, only the binding of the first paratope gives rise to a frequency shift in this experimental design and although most molecules bind two epitopes, a simple 1:1 binding kinetic equation can adequately describe the observed binding.

Data curves obtained at different antibody concentrations allow more accurate estimation of both the association and dissociation rate constants for PAM 4.7 binding to VAR2CSA DBL5ε either adsorbed to a polystyrene surface (Figure 2A) or covalently immobilized on a carboxyl surface (Figure 2B). Mass transport effects were initially estimated, and then minimized, by increasing flow rates. Surface density modulation was also used as a tool to explore avidity effects, as described below. Optimum flow rate was determined in each experiment. The fitting model also takes into account mass transport effects by adding a diffusion step. In Figure 2B, the association/dissociation curve for the binding of PAM 4.7 shows a fairly low binding strength (K_D _= *k*_*off *_/*k*_*on *_= 1.89 × 10^-4 ^M^-1^s^-1^/6.5 × 10^3 ^M^-1^s^-1 ^= 2.91 × 10^-8^M).

**Figure 3:**
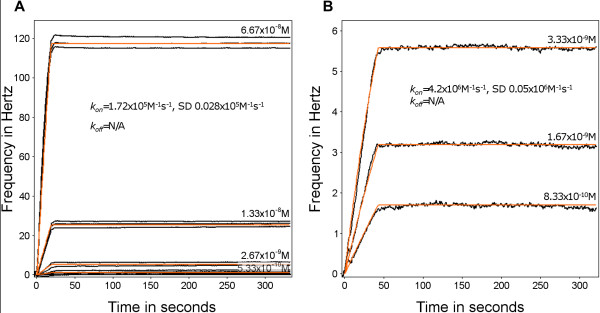
**Association and dissociation of a strongly-binding antibody with different surface densities of VAR2CSA DBL5ε**. The frequency shift (Hz) is shown as a function of time (s). Black lines represent data from different reactions, with the indicated concentrations of PAM 3.10 binding to VAR2CSA DBL5ε immobilized on a carboxyl surface. Red lines indicate the fitted curves, as described in the Methods. The rate constants calculated are given on the sensorgram. **A**. PAM 3.10, in flow, against a high density surface. **B**. PAM 3.10, in flow, against a low density surface. See Additional file 1 for surface antigen density calculations.

Figure 3 shows the binding of a second monoclonal antibody, PAM 3.10 to the DBL5ε domain of VAR2CSA. PAM 3.10 has both a higher *k*_*on *_(Figure 3A: 1.72 × 10^5^M^-1^s^-1^, SD 0.028 × 10^5^M^-1^s^-1^, Figure 3B: 4.2 × 10^6^M^-1^s^-1^, SD 0.05 × 10^6^M^-1^s^-1^) and a lower *k*_*off *_than PAM 4.7. In fact, the PAM 3.10 *k*_*off *_rate is too slow to be measured and there was no visible dissociation. This is a technical limitation of the biosensor as the instrument cannot measure very low dissociation rate constants (below around 10^-6^s^-1^, Attana AB, pers. comm.). Therefore in this experiment, an exact affinity constant cannot be calculated for the PAM 3.10 - antigen interaction. However, assuming k_off _< 10^-6^s^-1 ^the K_D _would be less than 2.38 × 10^-13^M. Thus, PAM 3.10 clearly binds more strongly than PAM 4.7 to the same immobilized PfEMP1 antigen fragment. A 10.000-fold difference in K_D _based on measurements under avidity conditions would roughly correlate to a 100-fold difference in K_D _based on measurements under affinity conditions.

### Analysing the avidity of anti-PfEMP1 antibodies by lowering the density of receptors

To maintain the antibody-in-flow approach while decreasing avidity effects that are apparent in the above experiments, the density of the immobilized PfEMP1 antigen on the chip surface was reduced. Ideally, this would also bring the *k*_*off *_of strongly binding antibodies into a detectable range. Figure 3 shows the sensorgram results of binding assays of PAM 3.10 reacting with the VAR2CSA DBL5ε, at different surface densities of immobilized antigen.

In Figure 3B, a low density surface (immobilization frequency shift of 18 Hz) was created to carry out a PAM 3.10 binding experiment under conditions less favourable to cross-linking than those used in Figure 3A, which used a high density surface (85 Hz frequency shift). From the immobilization frequency shifts observed and using Equation II (Additional file 1), 20 ng of mass were covalently bound onto the low-density surface chip, from 250 ng injected. During the creation of the higher density surface, 95 ng of protein antigen were covalently bound, from 2500 ng injected into flow (Figure 3A). The absorbed mass can be converted into number of molecules per surface. The low-density surface has 3.0 × 10^11 ^molecules and the high-density surface 1.4 × 10^12 ^molecules. The estimated distance between bound VAR2CSA DBL5ε molecules at low density was 9.8 nm (Additional file 2). At high density it decreased to 4.5 nm. However, the binding observed in Figure 3 indicates that even after reducing the mass of antigen immobilized on the surface, the *k*_*off *_remains outside the detectable range. The *k*_*on *_is increased from the value seen using the high density surface (Figure 3A, 1.72 × 10^5 ^M^-1^s^-1^) to 4.2 × 10^6 ^M^-1^s^-1^, *i.e*. approximately 25 fold. Given that further reductions in immobilized antigen mass leads to problems of markedly decreased sensitivity of specific detection of antibody binding from the flow, the alternative approach of attempting to measure single binding site affinities through the use of Fab fragments was employed.

### Measuring the affinity of single antibody binding sites using Fab fragments

**Figure 4:**
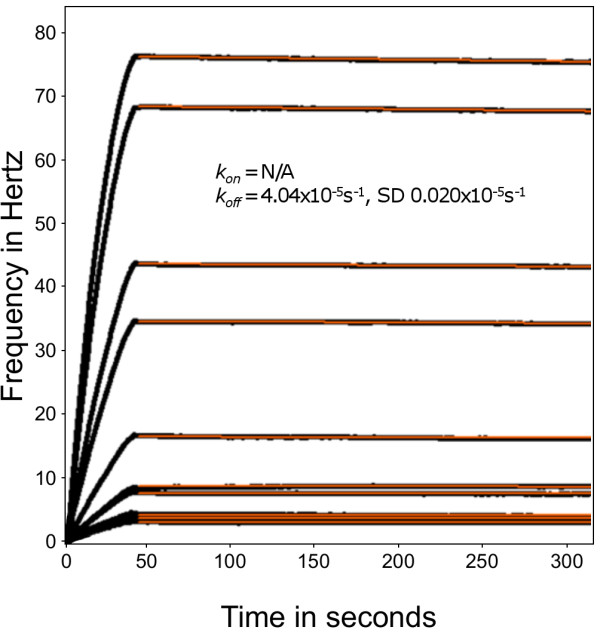
**The interaction of monovalent Fab fragment of PAM 3.10 with VAR2CSA DBL5ε**. Association and dissociation of the Fab fragments of PAM 3.10 to a high density of VAR2CSA DBL5ε on the sensor chip surface. The frequency change is shown as a function of time and the black lines show the data from binding experiments with different concentrations of the PAM 3.10 Fab fragment to the surface-bound protein. The red lines show the curve fit for the dissociation phase only. The dissociation rate constant calculated is given on the sensorgram.

Fab fragments produced by papain cleavage of IgG have a single binding site for antigen [36] and this simplifies analysis and permits direct determination of affinity. The data shown in Figure 4 are from experiments in which Fab fragments of PAM 3.10 were passed in flow over immobilized DBL5ε. To increase the strength of the analysis, the curves are fitted only to the dissociation phase, which now occurs, as predicted, in the measurable range with a rate *k*_*off *_= 4.04 × 10^-5^s^-1^, SD 0.02 × 10^-5^s^-1^.

The k_off _value of the Fab fragment describes the off-rate in a single antibody binding-site interaction, *i.e*. when only one 'arm' of the antibody binds. If an antibody binds without avidity being possible, by chance or due to the distance between epitopes, this would have an off-rate equal to the Fab-fragment off-rate. Avidity being approximately the product of affinities, while whole-antibody off-rates are not exactly Fab fragment off-rates squared, such Fab-derived values give an indication of where the whole-antibody off-rates lie. These quantitative comparisons of anti-PfEMP1 antibody binding strengths are in agreement with qualitative observations that PAM 3.10 is a better IFA staining antibody than PAM 4.7, in that it is useable at lower concentrations to give stronger staining and lower background *i.e*. a better signal-to-noise ratio.

### Modeling PfEMP1 packing onto the knob structures

Both immunofluorescence microscopy and binding kinetic analysis indicate that anti-PfEMP1 antibody binding is influenced by the molecular structures that pack and display these adhesion antigens on the IE surface. To attempt to understand the factors governing the molecular topology and packing of VAR2CSA on knobs, the packing density of the VAR2CSA PfEMP1 on the knob structures was modeled. Since both knobs and PfEMP1 are known to vary considerably in size the available surface area on representative knobs on BeWo-selected IE (FCR3) was measured. This line was used in the antibody assays and is the homologous system for the reagents.

**Figure 5:**
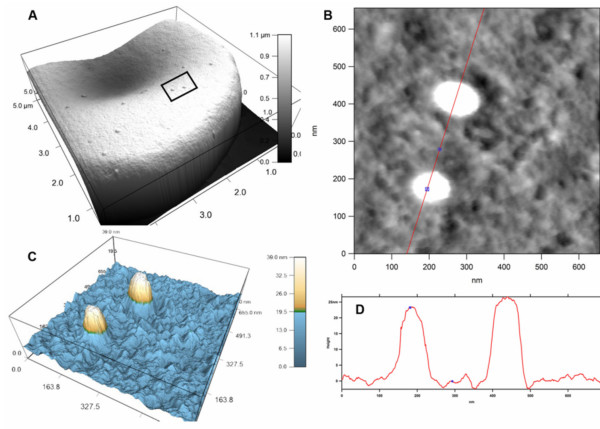
**AFM derived measurement of the dimensions of the knob structures on the IE membrane**. Force microscopy was carried out on unfixed, air dried IE. **A**. AFM tapping mode image of a single trophozoite-IE (FCR3). The boxed insert indicates the two knob structures analysed at higher resolution. **B**. Higher resolution scan data from the two knobs boxed in Figure 5A, bisected by the red sectioning line with the knob centre and a selected circumferential point indicated by the blue points. **C**. Two-colour enhanced three dimensional imaging of tapping-mode scan data. **D**. Topology measurements of the surface bisected by the red line shown in Figure 5B.

Although knobs were first detected and measured using electron microscopy, in more recent experiments AFM has been used to measure knobs under less denaturing conditions than those used in EM [37-41]. A simple, minimally denaturing AFM mounting methodology was used in these experiments, to scan unfixed IE dried onto microscope cover slips before being glued onto the magnetic AFM holder. Figure 5 shows the knobs on IE (FCR3), as detected using tapping mode AFM in air. The IE shown in Figure 5 were selected for BeWo cell and CSA adhesion and the expression of VAR2CSA antigen, and are recognized by both antibodies used in this study.

Figure 5A shows a scan of the early trophozoite surface and Figure 5B shows a higher resolution image of two knobs, around 250 nm apart on the membrane. Figures 5C and 5D show the line of section and contour cross-sections respectively. The proximal knob in the IE (FCR3) shown in Figure 5 is 120 nm in diameter and 24 nm high and its surface area is around 13,000 nm^2^ (Additional file 3).

The size and volume of the extracellular domains of VAR2CSA can also be estimated, based on extrapolation from atomic distances derived from recently published crystal structures of the VAR2CSA DBL3× domain [42,43]. With the *caveat *that the packing of the 6 DBL domains is unknown, for the purposes of this exercise in modelling PfEMP1 packing, we assume that VAR2CSA has a globular conformation, rather than a linear 'beads-on-a-string' arrangement of domains. The spherical volume of the extracellular 308 kDa of VAR2CSA will be around 840 nm^3 ^(Additional file 4). The diameter of this sphere is 12 nm and the area on the knob surface covered by a single such VAR2CSA configuration would be around 110 nm^2^(Additional file 4). If the knob shown in Figure 5 has a surface area of 13,000 nm^2^, a maximum of about 110 VAR2CSA molecules could be tightly packed onto this knob, at a density of around 8,000 molecules per μm^2 ^(Additional file 4).

It is important to stress that the above measurements and calculations indicate the *maximum *molecular densities of PfEMP1 proteins with a hypothetical globular conformation. In this study, the knob structures were visualized in AFM to provide molecular measurements in a homologous context and an easily conceptualized model to illustrate the parameters of the problem being analysed. How realistic these assumptions are in estimating PfEMP1 packing on the knobs, and in considering antibody cross-linking and malaria serology, is considered in the Discussion.

## Discussion

Under intense *Plasmodium falciparum *transmission, immunity to malaria is acquired in childhood. Disease and mortality are concentrated amongst infants and young children, although increased susceptibility to malaria occurs in pregnant women (reviewed in [44]). With successive pregnancies, some immunity to pregnancy-specific malaria is achieved [45]. Immuno-epidemiology [1,3,46] and direct experimentation [47], support a major role for antibody in natural immunity to malaria. Humoral immunity to pregnancy-associated malaria appears to require IgG antibodies against the VAR2CSA antigen, the receptor for chondroitin sulphate A on the placental syncytiotrophoblast. Our kinetic analysis of antibody binding to VAR2CSA uses monoclonal IgG1 antibodies from EBV-immortalized memory B cells from recently pregnant women living in a malaria endemic area of Africa [29]. The VAR2CSA DBL5ε antigen fragment binds to chondroitin sulphate A (CSA) [35,48,49] and both antibodies used in this study react with the surface of CSA-adhering IE in flow cytometry and bind to VAR2CSA DBL5ε-coated ELISA plates [29].

Measuring association and dissociation of these antibodies to surface-bound VAR2CSA, we have characterized representative low and high avidity antibodies. The high functional affinity of one antibody, PAM 3.10, was indicated by its very low, avidity-dependent, dissociation rate. The binding observed in Figure 3 indicates that even after reducing the mass of antigen on the surface by a factor of around 5, the *k*_*off *_for PAM 3.10 remained outside the detectable range. This observation is consistent with the Hexagon molecular spacing model calculation of inter-neighbour distances on the sensor surface and the distances measured between IgG1 antibody binding sites. Slow dissociation rates have been reported for other antibodies [25] and have also been proposed to be a manifestation of high avidity, caused by 'rocking' or switching between the two binding sites of a bivalent antibody [50,51].

Reducing the molecular density of antigen on the surface failed to increase the PAM 3.10 antibody dissociation rate to a measurable level. This is probably because cross-linking interactions between antigens 9.8 nm apart remains possible for human IgG1 antibodies. Their antigen-to-antigen 'reach' or sweep radius, as measured in co-crystallized antigen-antibody dimers, ranges from 14.2-17-1 nm [52]. However, abolishing bivalent binding by using Fab fragments did lead to a reduction in binding strength. The strength of monovalent binding dropped to a measurable level, comparable, although still greater, than the avidity of a bivalent, but relatively weak-binding antibody, PAM 4.7. This is consistent with the hypothesis that the unmeasurably low dissociation rate constants observed with PAM 3.10 are the result of avidity. Conversely, the lower avidity of PAM 4.7, even where bivalent interaction with the antigen is possible, indicates that its affinity is much lower than the affinity of the PAM 3.10 Fab fragment for the VAR2CSA antigen. This is also seen when comparing the avidity of PAM 4.7 to the affinity of PAM 3.10.

The antibody staining phenotypes of IE seen in Figure 1 confirm that surface reactive IgG binds PfEMP1 in discrete entities dispersed over the erythrocyte surface [20,21,53]. Since high avidity binding will play an important role in blocking parasite cytoadhesion and/or precipitating antibody dependent cellular cytotoxicity, it is important to know how easily IgG can cross-link surface PfEMP1. This requires consideration of molecular size, inter-molecular distances and the surface topology of the IE, where the erythrocyte membrane knobs display PfEMP1 [22,54-56]. The PfEMP1 intracellular domain binds knob proteins such as KAHRP and PfEMP3 and link PfEMP1 to the erythrocyte cytoskeleton [57,58] and transmembrane proteins such as Band 3 [59]. Knobs form at actin-spectrin-ankyrin junctions and inter-knob distances are clearly related to the regular inter-junction distances (100-200 nm) [60,61]. This makes antibody cross-linking of PfEMP1 between different knobs on the same erythrocyte by IgG1 (or even IgM) antibodies effectively impossible. By virtue of their cytoskeletal anchoring, knobs thus function as avidity reducing structures.

Since avidity strongly influences antibody binding, the question of *in vivo *access of cross-linking antibody paratopes to surface-exposed PfEMP1 epitopes on the same knob also arises. VAR2CSA is a large molecule with a total mass of 355 kDa, of which 308 kDa are in the six surface-exposed DBL domains. The topological parameters influencing intra-knob cross-linking and the establishment of high avidity binding will thus be knob surface area and density, PfEMP1 antigen size and the sweep radius between paratopes. Using EM, knob structures have been estimated to range from 70-150 nm in diameter and between around 10 and 40 nm in height [60,62]. Treating the 'average knob seen in EM' (*i.e*. in fixed cells prepared for EM, visualized under high vacuum) as sections of a spherical entity with diameter of 100 nm and height of 15 nm, their surface area can be calculated (Additional file 3) to be 8,600 nm^2^. For comparison, the surface area of a normal red blood cell is 140 μm^2 ^(1.40 × 10^7^nm^2^). The AFM-detected knob shown in Figure 5 has a surface area of 13,000 nm^2^. Assuming a globular structure for VAR2CSA, based on recent crystallographic measurements [43], an estimated maximum of 110 VAR2CSA molecules could be tightly packed onto a knob of the dimensions shown in Figure 5. The Figure 5 knob is a little bigger than the average found in IE (It/A4), but within the range observed in that AFM study [63].

However, a potentially major constraint on PfEMP1 packing density is the fact that knobs remain coated with intra-membranous particles (IMPs) [53,64] composed of host membrane proteins such as Band 3, glycophorin A & C, Rhesus proteins, CD47, and glucose transporters [65]. Unless the knob IMPs are composed of PfEMP1 without native host protein, which has not been observed, PfEMP1 must share the knob surface with host IMPs, thus further reducing its packing density. The circumferential zone around the knob is visibly depleted of IMPs and could potentially accommodate PfEMP1, in which case around thirty globular VAR2CSA molecules could be packed in a circumferential monolayer around a 120 nm diameter knob. In a rare crystallographic characterization of a membrane protein, a single subunit of rhodopsin (40 kDa) has been shown to occupy a unit volume of 7.5 nm × 4.5 nm × 3.0 nm [66,67]. These tightly packed photoreceptors have an estimated average packing density of 48,300 rhodopsin monomers per μm^2^, compared to our estimated maximum density of 8,000 VAR2CSA molecules per μm^2^.

Given the variability in knob size and the constraints on PfEMP1 packing density, it can be tentatively estimated that VAR2CSA-loaded knobs ranging in diameter between 80-130 nm, will contain around 10-80 of these large antigens. The lower end of these estimates is probably more realistic, particularly if VAR2CSA is more globular than rod-like, as proposed by a recent structural model [68]. The lower end of this estimate is close to a previous estimate of the number of PfEMP1 antigens on a knob, based on immuno-gold labeling of antibodies [53]. The presence of other parasite surface antigens on the knobs will further decrease the potential packing density of PfEMP1.

The density of knobs on the erythrocyte increases as the parasite develops [41,60] although their association with the regularly spaced cytoskeletal junctions determines their final density. Inter-knob distances of less than half the diameter of a knob are not observed [21,41,53,56,60,64,69]. Perhaps 1500-2000 knobs appear by the end of intra-erythrocytic development [41], although many fewer are often seen, *e.g*. in the young trophozoite surface shown in Figure 5. A maximum of 150-200,000 closely packed PfEMP1 molecules could thus be displayed on a heavily knobbed cell. Orders of magnitude fewer will be present on sparsely knobbed cells, irrespective of packing.

An established phenomenon in malaria serology, the serum-mediated agglutination of IE, requires cross-linking PfEMP1 molecules on separate erythrocytes [16,18]. *Ex-vivo*, serum antibodies cross-link IE *via *parasite VSAs (*e.g*. Figures 1G and 1H). Broadly agglutinating sera are not observed, indicating the high variability of such antigens. *Plasmodium falciparum *isolates from pregnant women are also rarely agglutinated by other malaria immune serum [70] and somewhat surprisingly, nor do plasma antibodies from malaria-exposed pregnant women readily agglutinate CSA adhesion-selected parasites [71]. It has been suggested that one reason for this is that high-affinity IgG binding to placental parasites inhibits the binding of potentially agglutinating IgM that is otherwise present in the sample [72]. The finding of very low dissociation rates with these IgG1 antibodies from VAR2CSA supports this hypothesis that IgG1 plays a tight-binding and agglutination-blocking role.

It also seems likely that high avidity antibodies primarily mediate intra- rather than inter-erythrocytic cross-linking. This is supported by the poorly agglutinating nature of the predominantly IgG immune sera isolated from multigravid, malaria-immune women in regions of holoendemic malaria [71]. Somewhat counter-intuitively, it appears that high avidity, strongly binding antibodies are less likely to mediate agglutination than low avidity antibodies, which by being less capable of intra-erythrocytic bivalent binding are more likely to have a paratope available to cross-link other IE.

## Conclusion

In summary, the significance of avid, bivalent binding for high-strength antibody binding to dispersed parasite surface antigens is shown. Molecular modeling of the PfEMP1-knob-antibody interaction indicates that the large size of PfEMP1 and the architecture of the knobs facilitate cytoadhesion yet may have an inhibitory effect on the avidity of antibody-PfEMP1 binding. Adhesion blocking vaccines should aim to induce strongly adhering, high avidity antibodies that do not dissociate readily from their target antigens. However, the association rate is probably less important in this context.

## Competing interests

The authors declare that they have no competing interests.

## Authors' contributions

LMJ and DEA conceived the main hypotheses and experiments, which were largely carried out by LMJ except for the atomic force microscopy experiments carried out by TH and the confocal microscopy by DB. LMJ performed the kinetic analysis and topological mathematical analyses and calculations. AS, LB, TD, LH and TGT produced antibodies, parasite lines and recombinant antigens and helped analyse the data. DEA and LMJ wrote the manuscript. All authors helped finalise the manuscript. All authors read and approved the final manuscript.

## Supplementary Material

Additional file 1 **Estimating molecular size and surface density**. Mathematical calculations estimating the size of a molecule based on its molecular mass, and the density of molecules on a surface. Click here for file

Additional file 2 **Topological models and calculations: the hexagon model for molecular distance**. Mathematical calculations determining the shortest distance between molecules on a surface assuming a distribution of molecules that maximizes the distances between molecules. Click here for file

Additional file 3 **Topological models and calculations: the area of spherical cross sections and the estimation of knob surface area**. Mathematical calculations estimating the surface area of a knob assuming the knob is a section of a sphere. Click here for file

Additional file 4 **A crystallographically aided estimate of the size of a VAR2CSA extracellular monomer**. Mathematical calculations estimating the size of VAR2CSA based on knowledge of the size of a single DBL gained from crystallography. Click here for file
