# Supplementary material for: The kinetics of antibody binding to Plasmodium falciparum VAR2CSA PfEMP1 antigen and modelling of PfEMP1 antigen packing on the membrane knobs
Source: Malar J. 2010 Apr 19;9:100. doi: 10.1186/1475-2875-9-100 (PMC2868858; doi:10.1186/1475-2875-9-100)
Supplement: Additional file 1 — Estimating molecular size and surface density. Mathematical calculations estimating the size of a molecule based on its molecular mass, and the density of molecules on a surface. [file 1475-2875-9-100-S1.PDF]

Additional file 1. Estimating molecular size and surface density

The molecular surface density calculation for the QCM biosensor's quartz crystal reaction surface was based on estimating the globular molecular radius (*R*) of the 40.1kDa DBL5ε domain fragment [1] and using the Sauerbrey equation for the relationship between change in quartz resonance frequency and change in mass [2]. The equation was modified to consider a reaction in water rather than the original assumption of a reaction in air [3]. This gave,

$$R = \left(\frac{3 \cdot Mw \cdot \nu}{4 \cdot \pi \cdot N_A} \right)^{\frac{1}{3}} \quad \textbf{(Equation I)}$$

Where ν is the partial specific volume of the molecule of interest, (volume per mass, taken to be 0.7317 ml x g^{-1}, a molecular constant calculated based on the composition of an average protein), and N_A is Avogadro's Constant (6.022 x 10^{23} mol^{-1}).

For the FCR3-derived VAR2CSA DBL5ε domain fragment, this gave a molecular radius of

$$R = \left(\frac{3 \cdot 40100 Da \cdot 0.7317 \frac{ml}{g}}{4 \cdot 3.1415 \cdot 6.022 \cdot 10^{23} mol^{-1}} \right)^{\frac{1}{3}} \approx 2.27 \cdot 10^{-7} cm = 2.27 nm$$

Change in mass is a linear function of the change in frequency [2]. Equation II then describes the change in mass on the surface of a crystal with one liquid-exposed surface.

$$\Delta m = \frac{\Delta f \cdot A \sqrt{\rho_q \mu_q}}{-2 \left(f_0 - f_0^{\frac{3}{2}} \sqrt{\frac{\eta_l \rho_l}{\pi \mu_q \rho_q}} \right)^2} \qquad \textbf{(Equation II)}$$

Where f_0 is the resonant frequency of the quartz crystal measured in Hz (10MHz), A is the piezo-electrically active crystal area ($0.25cm^2$), ρ_q is the density of quartz ($2.648g/cm^3$), μ_q is the shear modulus of quartz for an AT-cut crystal ($2.947x10^{11}g/(cm \cdot s^2)$), η_l is the dynamic viscosity of the liquid ($6.91x10^{-3}g/$ (cm x s) for water at $37^{\circ}C$), and ρ_l is the density of the liquid ($0.993g/cm^3$ for water at $37^{\circ}C$). Δm is calculated in grams.

Reference List

1. Rodbard D, Chrambach A: **Estimation of molecular radius, free mobility, and valence using polyacylamide gel electrophoresis.** *Anal Biochem* 1971, **40:**95-134.

2. Sauerbrey G: **Verwendung von Schwingquarzen zur Wägung dünner Schichten und zur Mikrowägung.** *Z Phys* 1959, **155:**206-215.

3. Kanazawa KK, Gordon JG: **Frequency of a quartz microbalance in contact with liquid.** *Anal Chem* 1985, **57:**1770-1771.
