# Supplementary material for: The kinetics of antibody binding to Plasmodium falciparum VAR2CSA PfEMP1 antigen and modelling of PfEMP1 antigen packing on the membrane knobs
Source: Malar J. 2010 Apr 19;9:100. doi: 10.1186/1475-2875-9-100 (PMC2868858; doi:10.1186/1475-2875-9-100)
Supplement: Additional file 2 — Topological models and calculations: the hexagon model for molecular distance. Mathematical calculations determining the shortest distance between molecules on a surface assuming a distribution of molecules that maximizes the distances between molecules. [file 1475-2875-9-100-S2.PDF]

Additional file 2. Topological models and calculations: the hexagon model for molecular distance

This model describes the minimum distance between two molecules on an immobilized surface assuming even distribution. The hexagon model places the molecules (red dots) in the corners of 6 equilateral triangles combined to form a hexagon. This has been formally proven to be the optimal method of stacking circles in two dimensions [1]. This model allows for a maximum area coverage of $\frac{1}{6}\pi\sqrt{3} \approx 0.907$ or 90.7%.

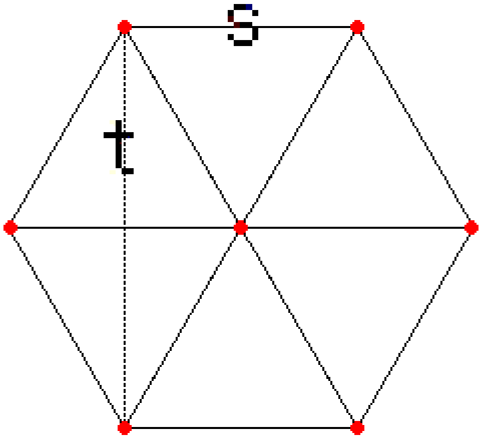

s = the side of the triangles, which is the length between the centres of two molecules.

t = the projected height of two triangles

Using the Pythagorean Theorem, t is expressed as a function of s as follows,

$$(2s)^2 = s^2 + t^2 \Rightarrow t = \sqrt{(2s)^2 - s^2} = s\sqrt{3}$$

Let A_d = surface area of the gold disc covering the quartz crystal and A_s = surface area of a square

By approximating a square to the disc where $A_d = A_s$, which is reasonable as the radius of the disc is much larger than the radius of a molecule, the problem can be reduced to a more easily solvable form. By removing every other molecule, the middle line of 3 molecules shown in the picture above, the problem is further reduced to a number of *t* x *s* squares in A_s, where each square corresponds to one molecule (again an approximation, but with an error on the scale of 10^{-9} %).

Let $m_{½}$ = half the number of molecules immobilized on the surface.

If A_s is divided by the area of a *t* x *s* square, the result will be the number of *t x s* squares which is equal to half the number of molecules immobilized, or in other words;

$$\frac{A_s}{s \cdot \sqrt{3} \cdot s} = m_{½} \Rightarrow s^2 = \frac{A_s}{m_{½} \cdot \sqrt{3}} \Rightarrow s = \sqrt{\frac{A_s}{m_{½} \cdot \sqrt{3}}}$$

Reference

1. Tóth LF: **On the Stability of a Circle Packing.** *Annales Universitatis Scientiarum Budapestinensis Sectio Mathematica* 1961,63-66.
