# Supplementary material for: The kinetics of antibody binding to Plasmodium falciparum VAR2CSA PfEMP1 antigen and modelling of PfEMP1 antigen packing on the membrane knobs
Source: Malar J. 2010 Apr 19;9:100. doi: 10.1186/1475-2875-9-100 (PMC2868858; doi:10.1186/1475-2875-9-100)
Supplement: Additional file 3 — Topological models and calculations: the area of spherical cross sections and the estimation of knob surface area. Mathematical calculations estimating the surface area of a knob assuming the knob is a section of a sphere. [file 1475-2875-9-100-S3.PDF]

Additional file 3. Topological models and calculations: the area of spherical cross sections and the estimation of knob surface area

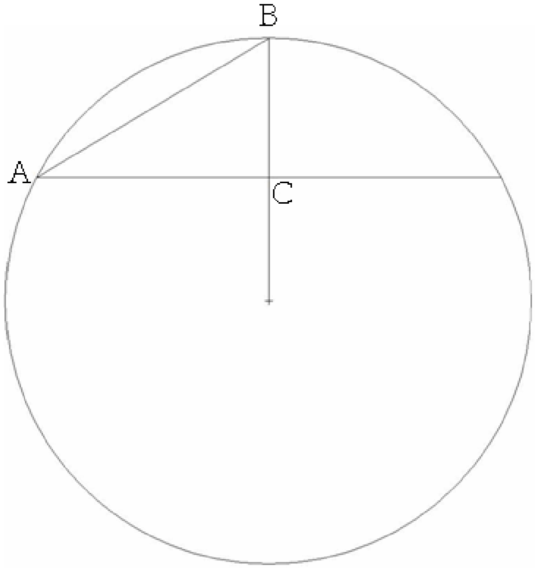

Archimedes' Theorem states that the surface area of a section of a sphere, defined by a plane perpendicular to the radius of the sphere, is equal to the surface area of a circle with radius equal to |AB|.

Given that a knob can best be considered to be approximately a section of a sphere and for a knob having a diameter of 120nm and a height of 24nm (as seen in Figure 5), |AB| can then be calculated using the Pythagorean Theorem.

$$|AB| = \sqrt{|AC|^2 + |BC|^2} = \sqrt{(60nm)^2 + (24nm)^2} \approx 64.6nm$$

This gives a surface area of:

$$A = \pi \cdot |AB|^2 \approx 13000nm^2$$

The estimated molecular area of the extracellular amino acids (2,500) of VAR2CSA, projected onto the knob surface is $64nm^2$, assuming the extracellular domain is globular. This gives a crude estimate, based solely on mass and the assumption of globularity, of the maximum number of VAR2CSA molecules that could be accommodated on an AFM visualized knob with surface area $13000nm^2$ of around 185 molecules/ knob.
