# Supplementary material for: The kinetics of antibody binding to Plasmodium falciparum VAR2CSA PfEMP1 antigen and modelling of PfEMP1 antigen packing on the membrane knobs
Source: Malar J. 2010 Apr 19;9:100. doi: 10.1186/1475-2875-9-100 (PMC2868858; doi:10.1186/1475-2875-9-100)
Supplement: Additional file 4 — A crystallographically aided estimate of the size of a VAR2CSA extracellular monomer. Mathematical calculations estimating the size of VAR2CSA based on knowledge of the size of a single DBL gained from crystallography. [file 1475-2875-9-100-S4.PDF]

Additional file 4. A crystallographically aided estimate of the size of a VAR2CSA extracellular monomer

The crystallographically determined unit size of a VAR2CSA DBL3X domain is 4.0nm x 5.0nm x 7.0nm [1,2] (and pers. comm. Dr. M. Higgins).

This gives a volume of 140nm^3.

Since the positioning of DBL domains relative to one another is unknown, for the purpose of these calculations it is assumed that the overall extracellular conformation of VAR2CSA is globular. It should be emphasized that while this assumption cannot be *absolutely* wrong, it is unsupported by any existing piece of data, crystallographic or otherwise.

The volume of the 6 DBL-type extracellular domains of VAR2CSA will be approximately 6 times that of a single DBL, so;

$$V_{VAR2CSA} = 6 \cdot V_{DBL3} = 6 \cdot 140nm^3 \approx 840nm^3$$

The volume of a sphere is

$$V = \tfrac{4}{3}\pi r^3$$

and the radius of a sphere of that volume is

$$r = \sqrt[3]{\frac{3V}{4\pi}} = \sqrt[3]{\frac{3 \cdot 840nm^3}{4 \cdot 3.1416}} \approx 6nm$$

The area on the knob surface covered by a VAR2CSA monomer would then be

$$A = \pi \cdot r^2 \approx 110 nm^2$$

Allowing for a *maximum* of

$$\frac{13000 nm^2}{110 nm^2} \cdot \tfrac{1}{6}\pi\sqrt{3} \approx 110 \frac{VAR2CSA molecules}{knob}$$

Reference List

1. Higgins MK: **Overproduction, purification and crystallization of a chondroitin sulfate A-binding DBL domain from a *Plasmodium falciparum var2csa*-encoded PfEMP1 protein.** *Acta Crystallogr Sect F Struct Biol Cryst Commun* 2008, **64:**221-223.

2. Higgins MK: **The structure of a chondroitin sulfate-binding domain important in placental malaria.** *J Biol Chem* 2008, **283:**21842-21846.
